# Supplementary material for: Resounding failure to replicate links between developmental language disorder and cerebral lateralisation
Source: PeerJ. 2018 Jan 8;6:e4217. doi: 10.7717/peerj.4217 (PMC5764032; doi:10.7717/peerj.4217)
Supplement: Supplemental Information 1 [file peerj-06-4217-s001.docx]

Supplemental Information

Exploratory analyses of sex differences

As reported in the main analysis, there was a small sex difference in fTCD LI. In the multilevel model with age and sex as fixed effects and twin pair membership as a random effect, sex was significant, *t* (198.45) = -2.24, *p* = .026; age was not, *t* (147.58) = 0.50, *p* = .617. Marginal mean LI for boys was 2.10 [SD = 2.78] and for girls was 1.32 [SD = 2.78]. It has been argued that language in females may be more bilateral than in males (McGlone, 1980), which this finding supports. However, the existence of a sex difference in lateralisation is controversial, and a meta-analysis of behavioural, structural and functional lateralisation for language did not reject the null hypothesis (I. E. Sommer, Aleman, Somers, Boks, & Kahn, 2008). Groen et al. (2012), who used the same fTCD task in a smaller sample of similarly-aged children as this study, did not find a significant sex difference, though there was a non-significant trend for boys to be more left-lateralised for language. Given that the effect size for the sex difference in this study was small (Cohen's *d* = 0.28), it is possible that Groen et al. (2012) did not have sufficient power to detect the effect.

Since there was a sex difference in fTCD LI at the full sample level, we re-ran multilevel models with sex as an extra fixed effect to explore whether DLD might show different relationships with atypical language lateralisation based on sex. The full models in this exploratory analysis therefore included the fTCD measures as the dependent variable and group (TD or DLD) and sex as fixed effects that were allowed to interact, with twin pair membership as a random effect.

In the model with the quantitative fTCD LI as the dependent variable, sex was significant, *t* (244.26) = -2.20, *p* = .029. There were no effects for group, *t* (256.03) = -0.13, *p* = .900, or the sex by group interaction, *t* (252.62) = 0.91, *p* = .365. This analysis indicates that there was no relationship between DLD and reduced laterality when boys and girls were grouped separately, echoing the absence of a relationship at the full sample level. See Table S1 for marginal means for the TD and DLD groups separately for boys and girls.

**INSERT TABLE S1**

In the model with percent change in blood flow in the left MCA as the dependent variable, group showed a significant effect, *t* (238.34) = 2.23, *p* = .027. The main effect of sex was non-significant, *t* (256.87) = 0.11, *p* = .915, as was the interaction, *t* (246.13) = -1.56, *p* = .119. The model for percent change in blood flow in the right MCA showed the same pattern of results as the model for the left MCA. Thus, group was significant, *t* (248.60) = 2.56, *p* = .011, while there were non-significant results for sex, *t* (258.96) = 1.27, *p* = .205, and the group by sex interaction, *t* (253.97) = -1.96, *p* = .051. Since TD and boy were the base factors against which the effects were compared, this analysis suggests that there was greater flow in both arteries specifically for boys with DLD. Table 6 shows marginal means for the TD and DLD groups by sex.

In the next analysis, we re-ran the model predicting fTCD categorical laterality and allowed sex to interact with group at each level of the dependent variable. There was no sex by group interaction for bilateral compared to left laterality, *p*MCMC = .212, though there was for right compared to left, *p*MCMC = .014. As can be seen in Table S2, this effect was driven by virtually all right lateralised girls being typically developing (24 out of 25). Only one had DLD.

**INSERT TABLE S2**

This exploratory analysis indicates that sex *may* have a role to play in the relationship between laterality and DLD. Interestingly, the risk for DLD was significantly lower for those who were right rather than left lateralised for language in girls compared to boys; only 1 out of 25 right lateralised girls met criteria for DLD, compared to 30-50+% of boys and girls across all other laterality categories. It is important to note that this finding was the product of exploratory analysis, and not predicted a priori. It also does not fit existing theories of laterality and neurodevelopmental disorders, and may be a false positive. Finally, if there is a sex-specific relationship between language lateralisation and DLD, we might expect to have found an interaction between group and sex in the model predicting quantitative LI as well; the preceding analysis indicates that we did not. The link between sex, laterality and DLD therefore awaits further study in a pre-registered design where the current results are used to formulate a priori predictions.

References

Groen M, Whitehouse AJO, Badcock NA, Bishop DV. 2012. Does cerebral lateralization

develop? A study using functional transcranial Doppler ultrasound assessing

lateralization for language production and visuospatial memory. Brain and Behavior

2(3):256-269 DOI 10.1002/brb3.56.

McGlone J. 1980. Sex differences in human brain asymmetry: a critical survey. Behavioral and Brain Sciences 3(2):215-227 DOI 10.1017/S0140525X00004398.

Sommer IE, Aleman A, Somers M, Boks MP, Kahn RS. 2008a. Sex differences in handedness, asymmetry of the planum temporale and functional language lateralization. Brain Research 1206:76-88 DOI 10.1016/J.BRAINRES.2008.01.003.
