# Supplementary material for: Resounding failure to replicate links between developmental language disorder and cerebral lateralisation
Source: PeerJ. 2018 Jan 8;6:e4217. doi: 10.7717/peerj.4217 (PMC5764032; doi:10.7717/peerj.4217)
Supplement: Table S1 [file peerj-06-4217-s002.docx]

|  | **Marginal Mean** | **Lower 95% CI** | **Upper 95% CI** |
| --- | --- | --- | --- |
| TD boys: LI | 2.17 | 1.44 | 2.90 |
| DLD boys: LI | 2.10 | 1.44 | 2.76 |
| TD girls: LI | 1.15 | 0.60 | 1.70 |
| DLD girls: LI | 1.75 | 0.86 | 2.63 |
| TD boys: Left flow, % change | -0.88 | -2.06 | 0.31 |
| DLD boys: Left flow, % change | 0.83 | -0.29 | 1.94 |
| TD girls: Left flow, % change | -0.80 | -1.74 | 0.15 |
| DLD girls: Left flow, % change | -0.86 | -2.33 | 0.62 |
| TD boys: Right flow, % change | -2.70 | -3.86 | -1.55 |
| DLD boys: Right flow, % change | -0.76 | -1.85 | 0.32 |
| TD girls: Right flow, % change | -1.79 | -2.70 | -0.88 |
| DLD girls: Right flow, % change | -2.03 | -3.47 | -0.60 |
