# Supplementary material for: Resounding failure to replicate links between developmental language disorder and cerebral lateralisation
Source: PeerJ. 2018 Jan 8;6:e4217. doi: 10.7717/peerj.4217 (PMC5764032; doi:10.7717/peerj.4217)
Supplement: Table S2 [file peerj-06-4217-s003.docx]

| **Language Laterality** | **Group** | **Boys** | **Girls** | **Total** |
| --- | --- | --- | --- | --- |
| Left | TD | 38 | 58 | 96 |
| Bilateral | TD | 11 | 18 | 29 |
| Right | TD | 7 | 24 | 31 |
| Left | DLD | 52 | 26 | 78 |
| Bilateral | DLD | 8 | 11 | 19 |
| Right | DLD | 9 | 1 | 10 |
